# Characteristics of medication advertisements found in US women’s fashion magazines

**DOI:** 10.15171/hpp.2017.06

**Published:** 2016-12-18

**Authors:** Jennifer Mongiovi, Grace Clarke Hillyer, Corey H. Basch, Danna Ethan, Rodney Hammond

**Affiliations:** ^1^Department of Epidemiology, Mailman School of Public Health, Columbia University, New York, NY, USA; ^2^Department of Public Health, William Paterson University, Wayne, NJ, USA; ^3^Department of Health Sciences, Lehman College, The City University of New York, New York, NY, USA; ^4^Department of Health and Nutrition Sciences, Montclair State University, Montclair, NJ, USA

**Keywords:** Advertisements, Direct-to-consumer advertising, Health communication, Magazines, Over-the-counter drugs, Women’s health

## Abstract

**Background:** Although prescriptions are dispensed at discretion of medical professionals, many pharmaceutical companies use direct-to-consumer (DTC) advertising to increase sales. Over-the-counter (OTC) medications are similarly marketed.

**Methods:** We examined the content of advertisements in 38 issues of 9 popular US women’s fashion magazines. We evaluated target audience, health condition, product availability, message appeal, target to females, and mention of potential side effects and benefits.

**Results:** Sixty total medication advertisements were identified, 58.3% (95% CI: 45.8, 70.8) for prescription products. In magazines targeted to non-Hispanic Whites, >65% of advertisements were for OTC medications whereas 80% (95% CI: 66.7, 94.5) of advertisements found in Black/Latina magazines were for prescription medications. The rational appeal was used most commonly in non-Hispanic White magazines (75.9%; 95% CI: 60.3, 91.5). Emotional appeal was featured more often in prescription advertisements magazines (60.0; 95% CI:43.8, 76.2) compared to OTC (8.0; 95% CI: -2.6, 18.6).

**Conclusion:** Although emotional appeal may be effective for selling medication to women, it often does not completely inform consumers of potential risks.

## Introduction

The average person living in the United States sees 360 advertisements each day through television, radio, internet, newspapers, and magazines.^1^ Included in this high volume of product advertisement are targeted strategies to persuade consumers to purchase both over-the-counter (OTC) and prescription medications. Unlike the purchase of OTC products, consumers are not the final decision makers when it comes to prescription medications.^2^ The ultimate choice of which medication is prescribed lies with the physicians. Traditionally, prescription medication has been marketed exclusively to prescribing physicians and pharmacists. To more actively involve the consumer in the choice of prescription medication and to augment sales by increasing consumer demand, most pharmaceutical companies now utilize direct-to-consumer advertising (DCTA).^3^

The US Congress has supported several initiatives in an effort to protect consumers with regard to DTCA. Before the regulation of print advertisements, broadcasted promotional advertisements for prescription medications were required to include the drug’s chemical and brand names, drug indication, and potential side effects.^4^ Furthermore, in 1997 the US Food and Drug Administration (FDA) released guidelines that held prescription medication advertisements targeted to both consumers and physicians to the same standards.^5^ These federal revisions allowed pharmaceutical companies to forgo inclusion of certain information in media advertisements by directing consumers to a physician, website, toll-free telephone number, or full print advertisement, which adversely may have contributed to an increase in DTCA in broadcast media (e.g. TV, radio).^6^ The legislation on print advertisements for prescription medications has remained unchanged since then and requires a brief summary containing information on who should not take the drug, when the drug should not be taken, possible serious side effects, and what can be done to lower the chances of side effects as well as less serious and frequently occurring side effects.^7^ Over-the -counter medication advertising (OTCA), regulated by the Federal Trade Commission, fall under different standards that require prior substantiation that an objective claim in the advertisement is true and that the advertisement is not misleading through misrepresentation or omitted information. Further, consumers must be informed about how to avoid harm, or how the benefit of the OTC medication outweighs consumer harm.^8^

Women specifically are often targeted by pharmaceutical companies and have been found to be more easily persuaded, accepting of claims made in advertisements, and trusting even after a trust-violation in comparison to men.^9,10^ Advertisements targeting emotions may have greater appeal to women and research literature suggests that women are more conscious of their emotions than men, putting greater value on their feelings.^11^ Emotional appeal can be used in advertising to establish perceived benefit of a medication without necessarily including the health benefit and risk information, resulting in a more positive perceived benefit of the product or brand.^12^ Women may also be a more profitable audience to market to since they are diagnosed with depression and anxiety at twice the rate as men and are more likely to have a chronic condition that requires medical attention.^13^ These factors in combination with the established fact that women use more prescription medications in a month than men make women a prime population to be targeted by DTCA and OTCA.^14^

Opinions on medication advertisements vary among marketing strategists, pharmaceutical companies, consumers and their prescribing physicians depending on whether the goal is monetary or health-oriented. Currently, the United States and New Zealand are the only countries that legally permit DTCA.^15,16^ Many consumers believe that DTCA is a resource that aids them in taking a more active role in their health care and help initiate conversations with doctors.^2^ So persuasive is DCTA that the majority of individuals who requested a medication from their doctor after having seen a prescription medication advertisement were given a prescription for that drug.^17^ Doctors, however, have often claimed that the advertised medication requested was not the most effective for a particular patient and report that DTCA encourages individuals to seek unnecessary treatment.^18^ These actions may be contributing to both over-diagnosis of conditions and over-prescribing of medications.^18,19^ The requested medications are often more expensive than alternative treatments since these products are marketed for profit and may not be covered under insurance.^20^ On the contrary, some studies have found that DTCA can trigger an individual to seek out medical help when they typically would not have.^21,22^

The influence of OTCA on consumers has been overlooked since intake of these medications is often considered minimal, normal, and does not require the recommendation and monitoring of intake by a health care professional.^23^ Prior studies have determined that unlike DTCA, OTCA has featured more content regarding the benefits of a particular products than the risk, omitting potentially dangerous health consequences,^24^ an observation confirmed in our analysis. Overall, less information is presented through OTCA than DTCA, even though the targeted consumers are the final decision makers for purchasing these medications.^2^ Few have hypothesized on the decision-making process of consumers, resulting in limited information on consumer attitudes advertisements and the overall impact of OTCA.^2,23^

The purpose of this study was to both enumerate and assess the DTCA and OTCA in women’s fashion magazines. Specifically, this study addressed differences found in magazines marketed to non-Hispanic White, Black, or Latina women and assessed the presence of marketing appeals, products marketed specifically to women, and legally required content for advertisement of prescription and OTC medications. Information learned from this investigation will provide insight into the content and style of advertisements found in women’s magazines that potentially influence women’s decisions to seek out various medications.

## Materials and Methods

US women’s fashion magazines were selected for this analysis because of the high readership among women aged 27 to 46. This group of women are more likely to have spouses and children that they make medical decisions for, representing the same audience targeted by manufacturers of pharmaceutical and OTC drugs.^25,26^ The choice of the individual fashion magazines reviewed was based on the readership characteristics publicized by each magazine in their on-line media kits, indicating a high level of readership within the targeted age range. A total of 99 issues of 14 popular US women’s fashion magazines were reviewed for medication advertisements.^27-37^ The selected issues were published between January and August, 2014. Magazines were grouped as those marketed predominantly to non-Hispanic White and those marketed to the African American and Latina audiences. The majority of magazines targeted a non-Hispanic white women (*Allure, Cosmopolitan, Elle, Girl’s Life, Glamour, Marie Claire, Seventeen, Teen Vogue, Vogue*) and five of these magazines were marketed specifically to a Black or Latina audience (*Cosmo Latina, Ebony, Essence, Jet, Latina*). All pages in each magazine were examined for medication advertisements.

A coding sheet was adapted from previous magazine analyses.^38,39^ Advertisements included were paid advertisements (versus products deemed “Editor’s Picks” or featured in editorials/articles) for any medication (prescription or OTC). Product advertisements placed on the magazine’s back cover (but not the front) were included. These advertisements were categorized on type of product advertised and those that featured either prescription or OTC medications were included in analysis. Advertisements related to weight loss, dietary supplements, and vitamins were not included in the current study as these products are considered more similar to special foods.^40^ A total of 60 advertisements were found in 38 issues from 9 different magazines (*Allure, Cosmopolitan, Ebony, Essense, Glamour, Latina, Jet, Marie Claire*, and *Vogue*). Medication advertisements were not found in magazines with a median readership age less than 32 years.

Advertisements were categorized by health condition,^25^ as well as if the medication was available by prescription or OTC. Health conditions addressed in advertisements included mental health (depression, nerves), chronic disease management (asthma, cholesterol, diabetes, hepatitis C, high blood pressure, multiple sclerosis),^41^ acute conditions (allergies, heartburn, headache/migraine, pain), cold/cough, birth control, physical appearance enhancement (facial injections, eyelash extensions, acne) and vaccines.

The content and message appeal of each advertisement was also determined. Potential message appeals included emotional (showing happiness or peace of mind) and rational appeals (providing information).^42^ Whether the producted advertised was being marketed as exclusively for women was also determined. Lastly, the presence or absence of clear documentation stating the potential side effects and benefits of the advertised prescription and OTC medication was assessed. The kappa for inter-rater reliability of these classifications was determined to be 87.0%, which was determined by recoding 10% of all magazines selected by random number generation.

We conducted descriptive analyses that included testing for associations between content and either intended audience (non-Hispanic White vs. Black/Latina) or product availability (prescription vs. OTC) using chi-square analysis. All analyses were performed using IBM SPSS (version 22). The institutional review boards at William Paterson University, Teachers College, Columbia University, and Lehman College do not review studies that do not involve human subjects research.

## Results

A total of 60 medication advertisements from nine popular US fashion magazines marketed to women were assessed (Table 1). The number of medication advertisements found in a given magazine ranged from 1 to 4, with a median of 1 advertisement (standard deviation [SD] = 0.78). Over 50% (51.7%; 95% CI: 39.1, 64.3) of advertisements appeared in magazines targeted to an ethnic audience (Blacks and Latinas) (51.7%; 95% CI: 39.1, 64.3). Overall, most advertisements were for prescription medications (58.3%; 95% CI: 45.8, 70.8) but differed significantly by the type of audience to which the product was marketed to. Nearly all of the advertisements found in Black and Latina magazines were for prescription drugs (80.6%; 95% CI: 66.7, 94.5) whereas 65.5% (95% CI: 48.2, 82.8) of advertisements in magazines targeted to non-Hispanic White women were for OTC products (*P* < 0.0001).

A total of 60 medication advertisements from nine popular US fashion magazines marketed to women were assessed (Table 1). The number of medication advertisements found in a given magazine ranged from 1 to 4, with a median of 1 advertisement (standard deviation [SD] = 0.78). Over 50% (51.7%; 95% CI: 39.1, 64.3) of advertisements appeared in magazines targeted to an ethnic audience (Blacks and Latinas) (51.7%; 95% CI: 39.1, 64.3). Overall, most advertisements were for prescription medications (58.3%; 95% CI: 45.8, 70.8) but differed significantly by the type of audience to which the product was marketed to. Nearly all of the advertisements found in Black and Latina magazines were for prescription drugs (80.6%; 95% CI: 66.7, 94.5) whereas 65.5% (95% CI: 48.2, 82.8) of advertisements in magazines targeted to non-Hispanic White women were for OTC products (*P* < 0.0001).

In this sample, the most common health conditions address in advertisements were for cold/cough (23.3%; 95% CI: 12.6, 34.0) and mental health (18.3%; 95% CI: 8.5, 28.1) (Table 1). Advertisements in magazines marketed to non-Hispanic White women followed a similar trend: cold/cough (37.9%; 95% CI: 20.2, 55.6), physical appearance enhancement (27.6%; 95% CI: 11.3, 43.4), and mental health (17.2%; 95% CI: 3.5, 30.9) medications. Significant differences were found in magazines marketed to Black or Latina women vs. those targeting non-Hispanic White women: the most common advertisements in magazines for Black or Latina women were for chronic disease management (25.8%; 95% CI: 10.4, 41.2), followed by birth control (22.6%; 95% CI: 7.9, 37.3) and mental health (19.4%; 95% CI: 5.48, 33.3) medications (*P *< 0.0001).

**Table 1:** Characteristics of magazine sample advertisements by targeted audience (n = 60)

	**Total No. (%) (95% CI)**	**Non-Hispanic White No. (%) (95% CI)**	**Black/Latina No. (%) (95% CI)**	***P*** ** value**
		29 (48.3) (35.7, 60.9)	31 (51.7) (39.1, 64.3)	
Availability of medication				<0.0001
Prescription required	35 (58.3) (45.8, 70.8)	10 (34.5) (17.2, 51.8)	25 (80.6) (66.7, 94.5)	
Over the counter	25 (41.7) (29.2, 54.2)	19 (65.5) (48.2, 82.8)	6 (19.4) (5.5, 33.3)	
Health condition				<0.0001
Mental health	11 (18.3) (8.5, 28.1)	5 (17.2) (3.5, 30.9)	6 (19.4) (5.48, 33.3)	
Chronic disease management	9 (15.0) (6.9, 24.0)	1 (3.4) (-3.2, 10.0)	8 (25.8) (10.4, 41.2)	
Acute conditions	9 (15.0) (6.9, 24.0)	4 (13.8) (1.3, 26.4)	5 (15.1) (2.5, 27.7)	
Cold/cough	14 (23.3) (12.6, 34.0)	11 (37.9) (20.2, 55.6)	3 (9.7) (-0.7, 20.1)	
Birth control	7 (11.7) (3.6, 19.8)	0 (0.0)-	7 (22.6) (7.9, 37.3)	
Physical appearance enhancement	8 (13.3) (4.7, 21.9)	8 (27.6) (11.3, 43.4)	0 (0.0)-	
Vaccines	2 (3.3) (-1.2, 7.8)	0 (0.0)-	2 (6.5) (-2.2, 15.2)	
Marketing appeal^a^				
Emotional appeal	23 (38.3) (26.0, 50.6)	6 (20.7) (6.0, 35.5)	17 (54.8) (37.3, 72.3)	0.007
Rational appeal	38 (63.3) (51.1, 75.5)	22 (75.9) (60.3, 91.5)	16 (51.6) (34.0, 69.2)	0.051

^a^ Groups not mutually exclusive.

The majority of advertisements used a rational message appeal to attract consumers (63.3%; 95% CI: 51.1, 75.5) (Table 1). Medication advertising that appealed to the emotions was observed more often in magazines targeting Black and Latina women (54.8%; 95% CI: 37.3, 72.3) compared to magazines geared toward non-Hispanic White women (20.7%; 95% CI: 6.0, 35.5) (*P *= 0.007). Findings suggest that advertisements in magazines targeted to non-Hispanic White women use more rational appeal (75.9%; 95% CI: 60.3, 91.5) compared to those in magazines for Black and Latina women (51.6%; 95% CI: 34.0, 69.2).

In comparing prescription medication advertisements to OTC, the emotional appeal was featured more often in advertisements for medications that required a prescription (60.0; 95% CI: 43.8, 76.2) than those that did not (8.0; 95% CI: -2.6, 18.6) (Table 2). Rational appeal was used in nearly all advertisements for OTC medications (92.0%; 95% CI: 81.4, 102.6) compared to less than half of advertisements for prescription medications (42.9%; 95% CI: 26.5, 59.3) (*P *< 0.0001). Almost all advertisements contained information on the benefits (96.7%; 95% CI: 92.2,101.2) and possible side effects (93.3%; 95% CI: 87.0, 99.6) of the medication advertised. OTCA (16.0%; 95% CI: 1.6, 30.4) was more likely than DTCA to omit potential side effects (0%; *P *= 0.014). OTC medication advertisements were also less likely to be marketed exclusively for women (12.0%; 95% CI: -0.7, 24.7) than prescription medications (48.6%; 95% CI: 32.0, 65.2) (*P *= 0.003).

**Table 2:** Advertisement content by prescription requirement (n = 60)

	**Total No. (%)**	**Prescription required No. (%)**	**OTC** **No. (%)**	***P *** **value**
		35 (58.3) (45.8, 70.8)	25 (41.7) (29.2, 54.2)	
Marketing appeal^a^				
Emotional appeal	23 (38.3) (26.0, 50.6)	21 (60.0) (43.8, 76.2)	2 (8.0) (-2.6, 18.6)	<0.0001
Rational appeal	38 (63.3) (51.1, 75.5)	15 (42.9) (26.5, 59.3)	23 (92.0) (81.4, 102.6)	<0.0001
Product exclusively for women				0.003
Yes	20 (33.3) (21.4, 45.2)	17 (48.6) (32.0, 65.2)	3 (12.0) (-0.7, 24.7)	
No	40 (66.7) (54.8, 78.6)	18 (51.4) (34.8, 68.0)	22 (88.0) (75.3, 100.7)	
Possible side effects				0.014
Yes	56 (93.3) (87.0, 99.6)	35 (100.0) -	21 (84.0) (69.6, 98.4)	
No	4 (6.7) (0.4, 13.0)	0 (0.0) -	4 (16.0) (1.6, 30.4)	
Benefits described				0.808
Yes	58 (96.7) (92.2,101.2)	34 (97.1) (91.5, 102.7)	24 (96.0) (88.3, 103.7)	
No	2 (3.3) (-1.2, 7.8)	1 (2.9) (-2.7, 8.5)	1 (4.0) (-3.7, 11.7)	

Abbreviation: Over-the-counter. ^a^Groups not mutually exclusive.

## Discussion

The majority of medications advertised to women were for products only available with a prescription. More DTCA was featured in magazines targeted to Black/Latina women than magazines targeting non-Hispanic White women. This may be explained by the greater number of advertisements for chronic disease management and birth control found in these magazines. Compared to magazines sold to Black or Latina women, magazines marketed to non-Hispanic White women contained significantly more advertisements for cold/cough medications and products for changing physical appearance, many of which are available OTC. Of particular interest were the differences in marketing appeals used in advertisements targeted to women of different ethnicities. Emotional appeal was more than twice as likely to be used in advertisements found in Black or Latina magazines while rational appeal was used 50% more frequently in non-Hispanic White magazines.

Similar to the findings of others,^43^ the greatest number of medication advertisements in our sample was found in magazines targeted to Black women. Research indicates that magazines targeted to Black women less often contain advertisements for joint pain and high cholesterol, however, over 25% of the advertisements found in magazines aimed at the ethnic market were for chronic disease management. The only advertisements found in magazines marketed specifically to Latina women were for acute conditions and cold/cough, which is consistent with findings demonstrating that Hispanic Americans use considerably fewer prescription products overall than non-Hispanic White Americans, regardless of health conditions.^44^ Given this lack of expenditure and that Hispanic Americans are the largest minority group in the United States, there currently exists a large, underutilized audience that companies may look to target.^45^ This is of particular concern since research has found that Hispanic Americans rely more on DTC advertising than non-Hispanic White Americans and are less skeptical of claims.^46^

This study was limited by the number of advertisements used in analysis. This limited sample size still reflected the audience targeted by manufacturers of pharmaceutical and OTC products based on age range. The convenience sampling strategy may have introduced selection biased based on availability of magazines. However, regardless of where the issues were purchased, the magazines marketed to non-Hispanic white, Black, and Latina women, respectively, had the highest circulation of fashion magazines in the United States within their targeted demographic group. Future studies should include magazines of various genres specifically targeted to women in order to improve sample size and to limit this potential bias.

## Conclusion

The present study found that women of different ethnic backgrounds are not targeted similarly by pharmaceutical companies through advertisements in women’s fashion magazines. While health conditions may vary between ethnic groups, differences still exist in message appeals and product availability. Black or Latina women are more likely to be targeted by pharmaceutical companies through emotional appeal than non-Hispanic White women. Additionally, prescription drugs were advertised mainly with emotional appeal while OTC medications featured rational appeal. Although this type of appeal may be effective for selling a product, it often does not completely inform consumers of potential risks. Future studies should expand on the differences to better understand the influence of both DTCA and OTCA on women. Additionally, further research should be done to evaluate the attitudes of consumers towards claims made in advertisements, especially those for medications that are not regulated by medical professionals. Understanding the influence of these types of advertisements on potential consumers can promote conversations between health care workers and patients to better understand possible risks.

## Funding

This was an unfunded analysis.

## Ethical approval

Not applicable

## Competing interests

None.

## Authors’ contributions

JM conceived the research hypothesis, performed analysis, and assisted in manuscript preparation. CHB, GCH, and DE designed the study and assisted in manuscript preparation. RH collected the data and assisted in manuscript preparation.

## Acknowledgements

None.
